# Whole-Tree Water Use Efficiency Is Decreased by Ambient Ozone and Not Affected by O_3_-Induced Stomatal Sluggishness

**DOI:** 10.1371/journal.pone.0039270

**Published:** 2012-06-18

**Authors:** Yasutomo Hoshika, Kenji Omasa, Elena Paoletti

**Affiliations:** 1 Graduate School of Agricultural and Life Sciences, The University of Tokyo, Tokyo, Japan; 2 Institute of Plant Protection, National Research Council, Sesto Fiorentino, Florence, Italy; University of Illinois, United States of America

## Abstract

Steady-state and dynamic gas exchange responses to ozone visible injury were investigated in an ozone-sensitive poplar clone under field conditions. The results were translated into whole tree water loss and carbon assimilation by comparing trees exposed to ambient ozone and trees treated with the ozone-protectant ethylenediurea (EDU). Steady-state stomatal conductance and photosynthesis linearly decreased with increasing ozone visible injury. Dynamic responses simulated by severing of a leaf revealed that stomatal sluggishness increased until a threshold of 5% injury and was then fairly constant. Sluggishness resulted from longer time to respond to the closing signal and slower rate of closing. Changes in photosynthesis were driven by the dynamics of stomata. Whole-tree carbon assimilation and water loss were lower in trees exposed to ambient O_3_ than in trees protected by EDU, both under steady-state and dynamic conditions. Although stomatal sluggishness is expected to increase water loss, lower stomatal conductance and premature leaf shedding of injured leaves aggravated O_3_ effects on whole tree carbon gain, while compensating for water loss. On average, WUE of trees exposed to ambient ozone was 2–4% lower than that of EDU-protected control trees in September and 6–8% lower in October.

## Introduction

Tropospheric ozone (O_3_) is an important phytotoxic air pollutant and is also recognized as a significant greenhouse gas [1]. Tropospheric O_3_ level has been continuously increasing since the first direct measurements in 1874 and its atmospheric concentration is now twice or more than in the pre-industrial age in the northern hemisphere [2]–[4]. Phytotoxic nature of O_3_ has been well known for decades [5]–[12]. Ozone concentrations recorded in rural areas are higher than those in the city [13] and thus O_3_ is now considered as the air pollutant with the highest damage potential to forests [14].

As the penetration of O_3_ through the cuticle can be considered as negligible [15], uptake through the stomata is a crucial factor for assessing the adverse effect of O_3_ on plants [16]–[20]. However, effects of O_3_ on stomatal responses are not straightforward, as both reductions and sluggish responses have been reported [21], [22]. Reductions of stomatal conductance occur when measurements are carried out under steady-state conditions [23]. Sluggishness has been reported during dynamic stomatal responses to fluctuating photosynthetic photon flux density (PPFD) [22], [24]–[27], vapor pressure deficit (VPD) [27], and severe water stress imposed by severing a leaf [26], [28]–[30]. Sluggish stomatal control over transpiration may increase water loss. Plants live in a fluctuating environment. A fast gas exchange response to rapid changes in the environmental stimuli is the key for successful plant adaptation and competition [31]. Because of climate change, forest ability of water control and carbon sequestration under O_3_ pollution is of rising importance [14].

Scalar and conceptual uncertainties still limit the current understanding of the basic physiological mechanisms that underline responses of forests to O_3_ [32]. The scalar uncertainties are due to transfer of results from seedlings in controlled environments to mature trees in the field, while the conceptual uncertainties are due to contrasting results about whole-tree water use responses to ambient O_3_ [32]–[35]. In contrast, there is a general agreement about O_3_ exposure as a factor of reduced tree carbon sequestration and biomass [36], although the results usually come from steady-state measurements of photosynthesis.

Ozone visible injury of leaves may be used as a clear and easily quantifiable proxy of O_3_ foliar damage and is the only method to assess O_3_ damage in the field [37]. Ozone visible injury has been investigated in many European and North American tree and herbaceous species, and partly validated under controlled conditions [38], [39]. There are few reports of relationship between stomatal conductance and O_3_ visible injury. After onset of O_3_ visible injury, significant reductions in steady-state leaf gas exchange were recorded for tree species in chamber experiments [40]–[42]. Omasa et al. (1981) did not report any correlation between visible injury and stomatal O_3_ uptake in a leaf [43]. Dynamic stomatal response was slower in injured leaves (20% injury) compared to control leaves (0% injury) for manna ash (*Fraxinus ornus* L.) [28].

Our main objectives were to improve our knowledge of steady-state and dynamic stomatal response to O_3_ visible injury in adult trees in the field, and to assess whole-tree water loss and carbon assimilation under ambient O_3_ impacts. Measurements were carried out in an O_3_-sensitive poplar clone (Oxford, *Populus maximoviczii* Henry *× berolinensis* Dippel) [44]. The amount of leaf injury per tree was experimentally manipulated by applying the O_3_-protectant ethylenediurea (EDU, N-[2-(2-oxo-1-imidazolidinyl)ethyl]-N’-phenylurea). EDU *per se* does not affect gas exchange [45] and has been widely used to prevent O_3_ visible injury and determine O_3_ effects in many plant species [39], [45]–[47].

## Materials and Methods

### Experimental Site and Plant Material

The study was carried out in an experimental field site located in central Italy (Antella: 43°44' N, 11°16' E, 50 m a.s.l., 14.7°C as mean annual temperature and 1233 mm as total annual precipitation in 2010). Forty root cuttings of the O_3_-sensitive Oxford clone were planted in two lines in 2007. Every week over the growing seasons 2008–2010, each tree was irrigated with 1 to 2 L of water (WAT, control line) or 450 ppm EDU solution (EDU, treated line), according to the successful application of EDU as soil drench to adult trees [48]. In 2010, the mean tree height was 2.9 m, and the mean stem diameter at breast height was 19 mm. Soil moisture was measured in the root layer (30 cm depth) by EC5 sensors equipped with an EM5b data logger (Decagon Devices, Pullman WA, USA). On average, soil moisture was 21.1±0.2% during the gas exchange measurements (September-October) and 24.5±0.1% during the growing season (April to October). The values were between field capacity (25.5%) and wilting point (17.5%) for this type of soil, i.e. sandy clay loam. Air temperature, relative humidity and precipitation were recorded by a 110-WS-16 modular weather station (NovaLynx corp., Auburn CA, USA). Average vapor pressure deficit during daylight hours and total precipitation were 1.02 kPa and 197 mm in September to October and 1.42 kPa and 625 mm from April to October, respectively. Ozone concentrations were continuously recorded at canopy height (2.0 m) by an O_3_ monitor (Mod. 202, 2B Technologies, Boulder CO, USA). The AOT40 value (accumulated exposure above a threshold concentration of 40 ppb during daylight hours) during the growing season (April to October) was 25.8 ppm⋅h and the maximum hourly O_3_ concentration reached 118 ppb.

### Assessment of Ozone Visible Injury

Ozone visible injury occurred as dark stippling on the upper leaf surface since early September 2010. The injury was identified as O_3_-like because it was missing in shaded leaves and more severe in older than in younger leaves [38]. The symptoms were similar to those caused by ambient O_3_ in *Populus nigra* [42]. In September (22^th^ to 28^th^) and October (23^th^ to 28^th^), all 9502 leaves from five trees per treatment (WAT and EDU) were counted and assigned to 5%-step injury classes by the same two observers. Photoguides quantifying visible injury (0∼100%) by image analysis processing were used [38], [39]. Pest, pathogen and mechanical injury occurred in both EDU and WAT trees and was assessed to be <5% of total leaves. Leaves for measurements of gas exchange showed O_3_ visible injury only and were evaluated on a 1%-step basis.

### Measurement of Steady-state and Dynamic Gas Exchange

Fully expanded sun leaves (medium size) with visible injury from 0% to 50% at set positions from the terminal shoot (5th to 16th) of WAT trees were measured in clear sky days of September and October 2010 between 10∶00 and 15∶00 CET. Preliminary measurements did not show significant differences in gas exchange of healthy leaves, i.e. without visible ozone injury, at those set positions. Gas exchange was measured with a portable infra-red gas analyzer (CIRAS-2 PPSystems, Herts, UK), equipped with a 2.5 cm^2^ leaf cuvette which controlled leaf temperature (24°C), leaf-to-air vapour pressure deficit (1.0 kPa), saturating light (1800 µmol m^−2^ s^−1^) and CO_2_ concentration (375 ppm). Steady-state light-saturated photosynthesis (A_max_), stomatal conductance to water vapor (g_s_) and transpiration were measured in 41 leaves from WAT trees.

**Figure 1:**
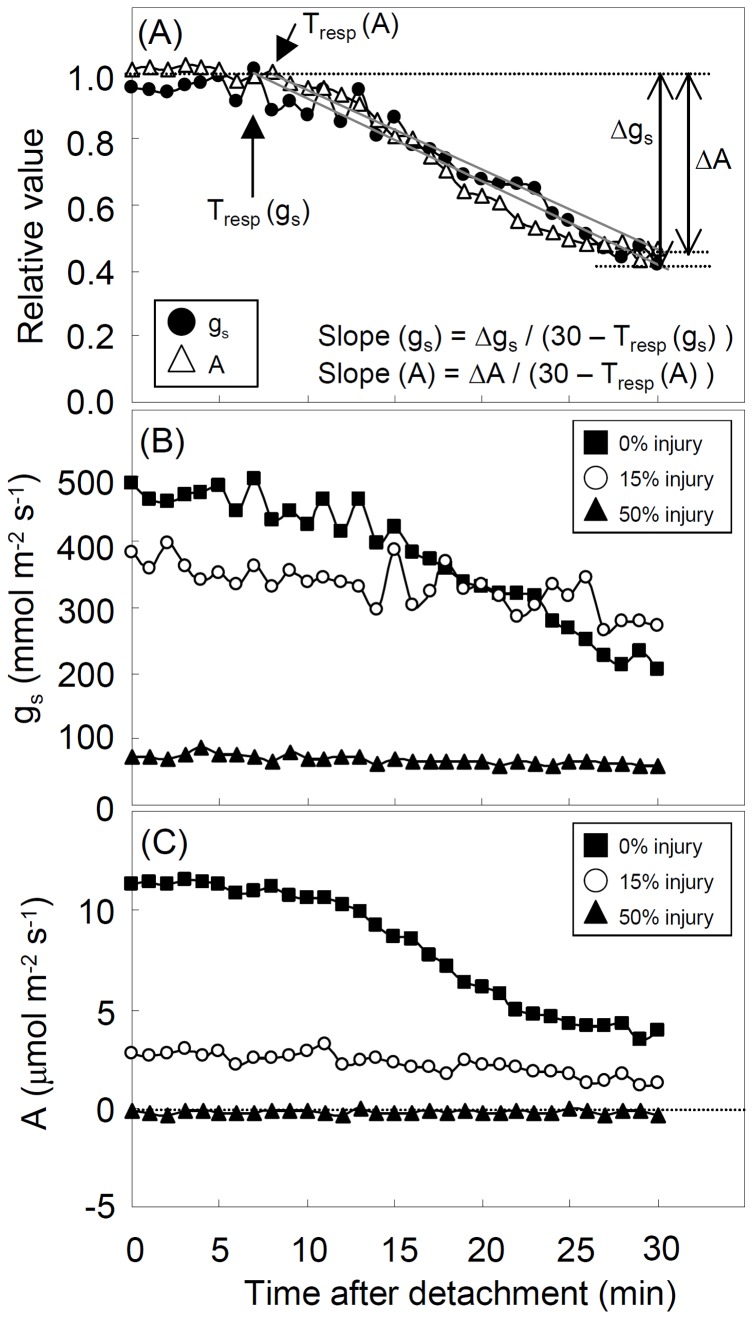
Examples of dynamic response of g_s_ and A_max_ after detachment of the leaf (A: calculation of the dynamic parameters in a leaf with 0% visible injury, B: time courses of absolute values in g_s_, C: time courses of absolute values in A). Δg_s_ and ΔA show the range of g_s_ and A_max_ variation, respectively, over 30 min from the leaf severing. T_resp_ (g_s_) and T_resp_(A) show the time to start decrease of g_s_ and A_max_, respectively. Slope(g_s_) and Slope(A) show the rate of decrease for g_s_ and A_max_, respectively, over 30 min.

Dynamic measurements were carried out for 21 leaves from WAT trees. When both g_s_ and A_max_ reached equilibrium under constant light at 1800 µmol m^−2^ s^−1^, the leaf petiole was severed with a sharp scalpel, similar to the methodology in Paoletti (2005) [26]. The data were logged at 1 min intervals for 30 min after severing. As the absolute value of g_s_ and A_max_ varied among individual leaves, relative g_s_ and A_max_ were expressed as a percentage of the average of the last 5 points at equilibrium, i.e., just before leaf severing. The following parameters were estimated based on fittings of two linear lines to minimize the root mean square error between measured and predicted values for g_s_ or A (Figure 1A): range of relative g_s_ decrease at 30 min after severing, Δg_s_; time to start g_s_ decrease, T_resp_ (g_s_); rate of g_s_ decrease at 30 min from severing, Slope(g_s_)  =  Δg_s/_(30– T_resp_ (g_s_)); range of relative A_max_ decrease at 30 min after severing, ΔA; time to start A_max_ decrease, T_resp_ (A); rate of A_max_ decrease at 30 min from severing, Slope(A)  =  ΔA/(30– T_resp_ (A)).

After measurements, the leaf area was measured by means of a leaf area meter (AM300, ADC, Herts, UK) for assessing a relationship between leaf size and the variation of g_s_ in single leaves. We hypothesized that the water content of a leaf may depend on leaf size and affect g_s_ response.

### Tree Level Modeling

To assess effects of O_3_ visible injury on leaf gas exchange at tree level, we constructed a simple model to scale up from single–leaf steady-state and dynamic gas exchange. The model was applied to the five trees per treatment (WAT and EDU) whose leaves were counted and assigned to a 5%-step visible injury class. Steady-state leaf water loss and photosynthesis at tree level, i.e. W_loss_: mol H_2_O tree^−1^ s^−1^, and A_tree_: µmol CO_2_ tree^−1^ s^−1^, were estimated as follows:
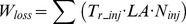
(1)

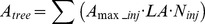
(2)where T_r_inj_ and A_max_inj_ are transpiration rate (mmol m^−2^ s^−1^) and photosynthesis (µmol m^−2^ s^−1^), respectively, at 1800 µmol m^−2^ s^−1^ constant light for leaves showing O_3_ visible injury. N_inj_ is the number of leaves in each 5%-step injury class. LA is the average leaf area per leaf (0.003 m^2^ leaf^−1^), calculated from subsamples of 30 randomly collected leaves per tree.

Whole-tree leaf water loss and carbon assimilation under the severe water stress simulated by severing a leaf (W_loss_st_: mol H_2_O tree^−1^ s^−1^, and A_tree_st_: µmol CO_2_ tree^−1^ s^−1^) were estimated by the following equations:
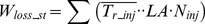
(3)

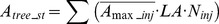
(4)where 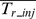 is the average transpiration rate (mmol m^−2^ s^−1^) and 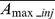 is the average photosynthesis (µmol m^−2^ s^−1^) at 1800 µmol m^−2^ s^−1^ constant light during the 30 min after severing a leaf with O_3_ visible injury.

### Statistical Analysis

Effects of O_3_ visible injury on steady-state leaf gas exchange and dynamic responses after severing a leaf were tested with a regression analysis. Correlation between variables of dynamic stomatal response was tested. Two-way analysis of variance (ANOVA) was used to assess the effects of measuring month and EDU treatments on number of leaves, ozone visible injury and gas exchange parameters at whole tree level. Differences among means were tested by Tukey HSD test. Percents were arcsine square root transformed prior to analysis. Data were checked for normal distribution (Kolmogorov-Smirnov D test) and homogeneity of variance (Levene’s test). Results were considered significant at p<0.05. All statistical analyses were performed with STATISTICA software (6.0, StatSoft Inc., Tulsa, OK, USA).

## Results

### Number of Leaves and Ozone Visible Injury

**Figure 2:**
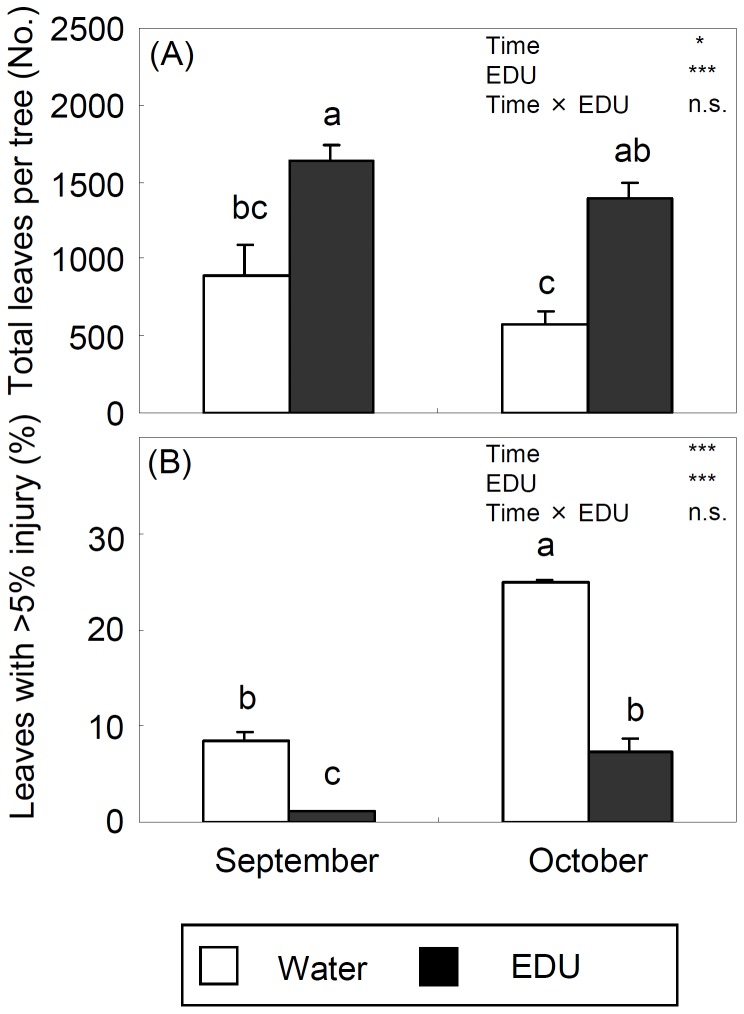
Total number of leaves (A) and percentage of ozone injured leaves (more than 5% of injured surface) (B) per tree (+SE) (WAT: water treated plants; EDU: EDU treated plants). * and *** denote significance at the 5% and 0.1% level, respectively; n.s. indicates no significance. Different letters above the bars indicate significant differences among bars (Tukey HSD test, P<0.05, n = 5 trees).

In September, EDU trees had 83% more leaves per tree than WAT trees (Figure 2A). In October, leaf abscission had progressed faster in WAT trees (−36% of leaves relative to September) than in EDU trees (−15%), resulting in EDU trees showing significantly more leaves (+144%) than WAT trees. The percentage of injured leaves (>5% of visible injury) was significantly higher in WAT trees than in EDU trees in both September and October (Figure 2B). In October, the percentage of injured leaves was 3.13 and 7 times higher than in September in WAT and EDU trees, respectively.

### Steady-state g_s_ and A_max_

**Figure 3:**
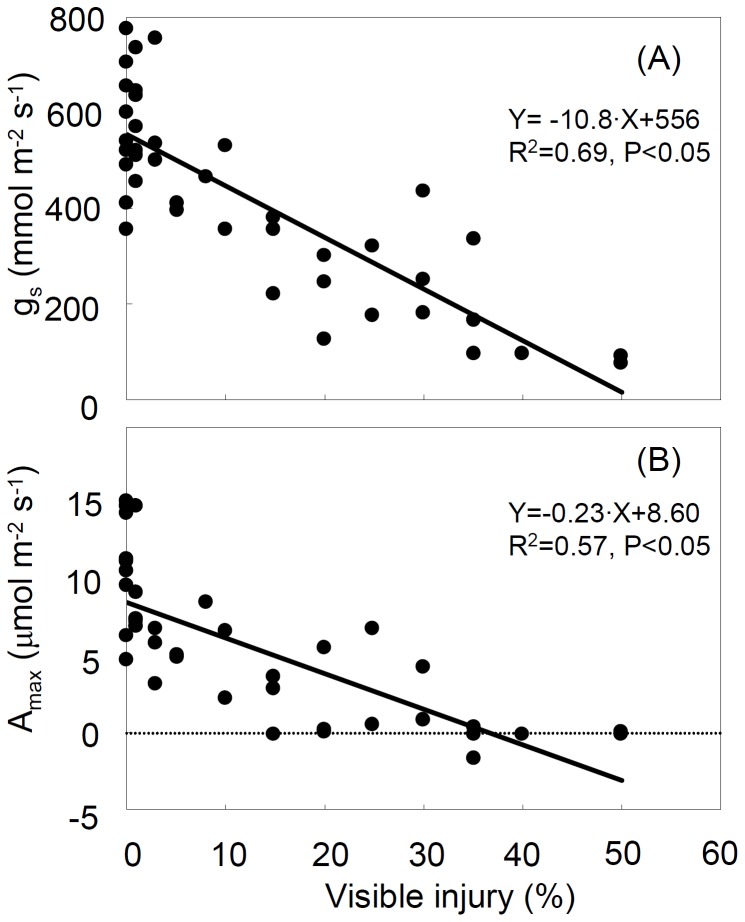
Relationships between steady-state leaf gas exchange (A: stomatal conductance (g_s_), B: light-saturated photosynthesis (A_max_)) and visible ozone foliar injury.

Steady-state leaf gas exchange for both g_s_ and A_max_ decreased with increasing O_3_ visible injury (Figure 1, 3). In healthy leaves (0% injury), g_s_ was 400 to 800 mmol m^−2^ s^−1^ whereas it was less than 100 mmol m^−2^ s^−1^ in leaves with 50% injury (Figure 3A). Leaves with higher injury (>50% injury) were tested but did not show a measurable g_s_. In control leaves, A_max_ was 5 to 15 µmol m^−2^ s^−1^, and dropped to around 0 µmol m^−2^ s^−1^ in leaves with more than 35% injury (Figure 3B).

### Variation of g_s_ and A_max_ after Detachment of a Leaf

After detachment of a leaf, two phases of gas exchange response were observed (Figure 1A): no response until T_resp_ and then a linear decrease. The magnitude of change in g_s_ at 30 min after severing a leaf (Δg_s_) did not depend on leaf size (data not shown: R^2^ = 0.02, p = 0.537) and thus on the total water content of a leaf.

**Figure 4:**
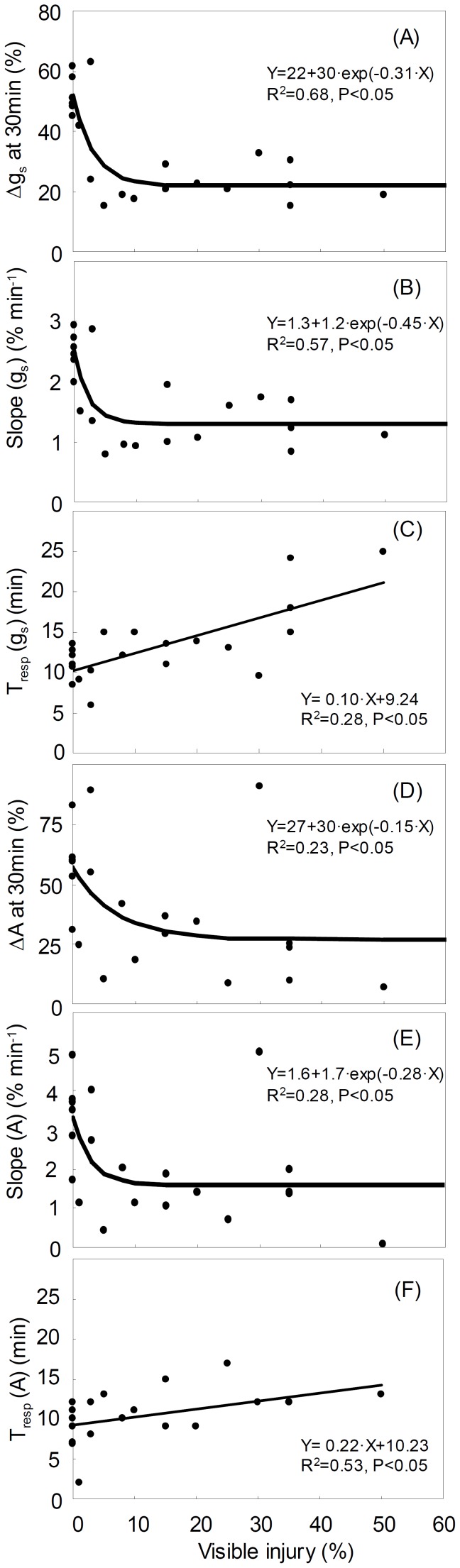
Relationships between visible ozone foliar injury and dynamic response of stomatal conductance (g_s_) and photosynthesis (A_max_) over 30 min after leaf severing (A: Δg_s_ at 30 min; B: Slope(g_s_); C: T_resp_ (g_s_); D: ΔA; E: Slope(A); F: T_resp_ (A)).

Figure 4 shows the relationships between O_3_ visible injury and dynamic response of g_s_ and A_max_. Δg_s_ showed a non-linear response to O_3_ visible injury (Figure 4A). It sharply decreased from 45–60% in healthy leaves (0% injury) to 15–30% in leaves with >5% visible injury. Slope(g_s_) sharply decreased from 2.5–3.2% min^–1^ in healthy leaves (0% injury) to 0.8–1.8% min^–1^ in leaves with >5% visible injury, and did not vary in leaves with 5–50% of injury (Figure 4B). The response time to start stomatal closing (T_resp_ (g_s_)) was linearly correlated to O_3_ visible injury (Figure 4C). T_resp_ (g_s_) increased from about 10 min in healthy leaves to >13 min in leaves with >20% injury. The magnitude of decrease in photosynthetic rate (ΔA) sharply decreased from about 55% in healthy leaves to about 25% in leaves with >5% visible injury (Figure 4D). Slope(A) sharply decreased from about 3.3% min^–1^ in healthy leaves to about 1.6% min^–1^ in leaves with >5% visible injury (Figure 4E). There was a linear relationship between the response time to start decrease of photosynthesis (T_resp_ (A)) and O_3_ visible injury (Figure 4F). T_resp_ (A) increased from 5–13 min in healthy leaves to 25 min in a leaf with 50% injury. Table 1 shows correlation between the A_max_ and g_s_ variables obtained from dynamic response after severing of a leaf. The magnitude of change in A_max_ (ΔA) increased with increasing Δg_s_. The rate of reduction in A_max_, i.e. Slope(A), was positively correlated with Slope(g_s_). The response times to start decrease of A_max_ and g_s_, i.e., T_resp_, were not significantly correlated, although they showed a statistical tendency to a positive correlation (p<0.1).

**Table 1:** Correlation between A_max_ vs. g_s_ variables obtained during the dynamic response to severing of a leaf (Δ: magnitude of change in A_max_ and g_s_ over 30 min from the leaf severing; T_resp_: time to start decrease in A_max_ and g_s_ after severing a leaf; Slope: rate of A_max_ and g_s_ decrease).

Parameter	Pearson coefficient	Level of significance
Δ	0.626	0.002**
Slope	0.622	0.003**
T_resp_	0.371	0.098 n.s.

** denotes the significance at 1% level; n.s. indicates no significant correlation.

### Carbon Assimilation and Water Loss at Tree Level

**Figure 5:**
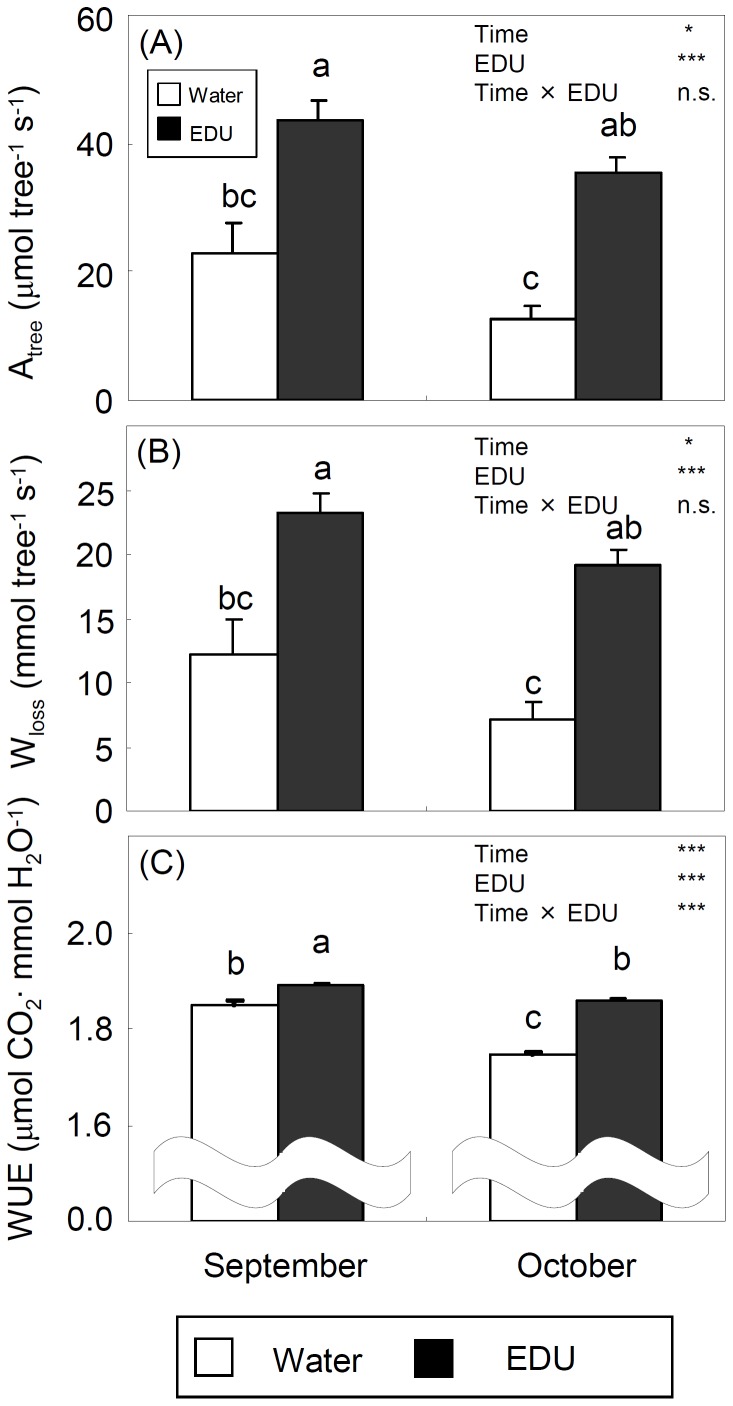
Estimated steady-state carbon assimilation (A: A_tree_), water loss (B: W_loss_) and instantaneous water use efficiency expressed as A_tree_/W_loss_ (C: WUE) at tree level (+SE) (WAT: water treated plants; EDU: EDU treated plants). * and *** denote significance at the 5% and 0.1% level, respectively; n.s. indicates no significance. Different letters above the bars indicate significant differences among bars (Tukey HSD test, P<0.05, n = 5 trees).

**Figure 6:**
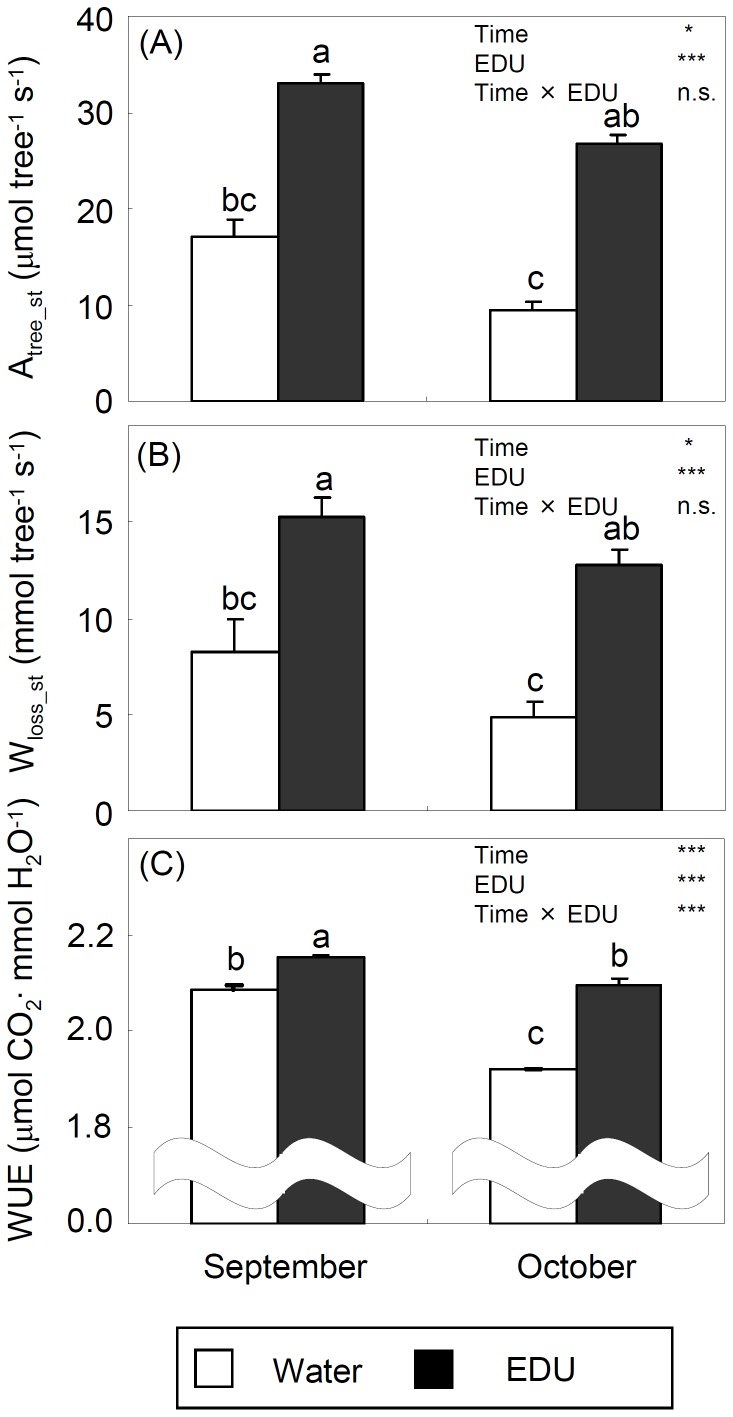
Estimated carbon assimilation (A: A_tree_st_), water loss (B: W_loss_st_) and instantaneous water use efficiency expressed as A_tree_st_/W_loss_st_ (C: WUE__st_) at tree level under severe water stress imposed by severing a leaf (+SE) (WAT: water treated plants; EDU: EDU treated plants). * and *** denote significance at the 5% and 0.1% level, respectively; n.s. indicates no significance. Different letters above the bars indicate significant differences among bars (Tukey HSD test, P<0.05, n = 5 trees).

In September, A_tree_ and W_loss_ were significantly lower in WAT trees, being half of the values in EDU trees (Figure 5A–B). In October, the difference between WAT and EDU trees became even larger. Whole-tree water use efficiency (A_tree/_W_loss_) at steady-state was significantly higher in EDU trees than in WAT trees both in September and October (Figure 5C). WUE decreased over time, but the decrease was larger in WAT (−6%) than in EDU trees (−2%), resulting in a significant Time x EDU interaction. Both in September and October, both A_tree_st_ and W_loss_st_, i.e. whole-tree carbon assimilation and water loss under the simulated severe water stress, were significantly lower in WAT trees (Figure 6A–B), similarly to the results from steady-state measurements (Figure 5A–B). Whole-tree instantaneous water use efficiency, expressed as A_tree_st/_W_loss_st_, was significantly higher in EDU trees than in WAT trees both in September and October (Figure 6C). Again, the decrease of WUE_st over time was larger in WAT (−8%) than in EDU trees (−3%).

## Discussion

According to previous reports in different species [28], [40]–[42], the steady-state measurements indicated that g_s_ and A_max_ linearly decreased with increasing leaf visible injury in the O_3_-sensitive Oxford clone (Figure 3). A_max_ dropped to around 0 µmol m^−2^ s^−1^ in leaves with more than 35% injury and g_s_ was not measurable in leaves with more than 50% injury. In a previous field study, leaves of manna ash with 20% visible injury showed a 33% reduction in g_s_ and A_max_ relative to healthy leaves (measurement in September) [28]. The result of the present study showed a larger reduction in g_s_ (about 39%) and A_max_ (about 54%) of 20% injured leaves, suggesting effects of O_3_ visible injury on gas exchange are species-specific. Paoletti et al. (2009a) suggested that the modifications of stomatal conductance in O_3_ injured leaves were driven by the structural alterations found in the mesophyll rather than by structural changes in stomata or other epidermal cells [28]. Omasa et al. (1981) suggested that stomatal opening in leaves with O_3_ visible injury varied with changes in the pressure balance between guard cells and epidermal cells caused by the water-soaking of epidermal cells [43]. The most likely changes, however, are due to photosynthetic impairment [21], [49].

When analyzing dynamic g_s_ response to severing of a leaf, stomata of injured leaves were shown to be slower than those of healthy leaves in responding to the closing signal (T_resp_ (g_s_)) and in the rate of closing (Slope(g_s_)) (Figure 4B–C). These combined effects translated in a lower ability of injured leaves to close stomata, i.e. in a lower Δg_s_ than healthy leaves, resulting in a sluggish stomatal control over water loss. In a previous study, Paoletti et al. (2009a) also reported a slower response of stomata to severing in leaves of manna ash with O_3_ visible injury [28], even though only leaves with 0% and 20% injury were compared. Here, we compared leaves with a range of O_3_ visible injury, i.e. from no injury (control) until a measurable g_s_ was recorded (∼50% injury) and showed that Δg_s_ decreased sharply above 5% injury and did not change any more (Figure 4A).

Literature results highlight several mechanisms by which O_3_ may induce sluggishness. Omasa (1990) reported a slight increase in permeability of epidermal cell membranes and alteration of the osmotic pressure after O_3_ exposure, that may modulate a balance in turgor between guard and subsidiary cells [50]. Vahisalu et al. (2010) found that Ca^2+^-dependent signaling and O_3_-induced stomatal movements were independent, and highlighted a temporary desensitization of the guard cells due to blocking of the K^+^ channels [51]. Another cause of sluggishness may be O_3_-induced lower rates of transpiration in which leaves take longer to perceive the same change in water status following petiole excision [26], [28]–[30] or light variation [22], [26]. All the above mechanisms, however, cannot explain the non-linear response of Δg_s_ to visible injury observed in the present study. Ozone may also delay stomatal responses by stimulating ethylene production and reducing stomatal sensitivity to ABA [52]. Ethylene production is known to increase with increasing O_3_ visible injury [53], [54]. In tomato plants, concentration of ACC (1-aminocyclopropane-1-carboxylic acid), a precursor of ethylene, increased when visible injury reached 5% and remained constant until the maximum injury recorded in the experiment, i.e. 35% [55]. A sharp rise of ethylene emission as soon as visible injury reaches 5% and a constant emission over this threshold would explain why Δg_s_ decreased sharply above 5% injury and did not change any more when injury was >5% (Figure 4A). Tuomainen et al. (1997) also showed that ethylene emission from detached leaves was enhanced fourfold in ozone-treated plants, while no changes were observed in control leaves that were similarly cut at the petiole [55].

Sluggish A_max_ responses with increasing O_3_ visible injury were also found in the measurements of dynamic leaf gas exchange (Figure 4D–F). The response of A_max_ was similar to that of g_s_ after severing a leaf (Figure 1), i.e. no response until T_resp_ and then a linear decrease during stomatal closure. Although the response time to start reduction of A_max_ was not significantly correlated with the response time to closing stomata, the magnitude and rate of reduction in A_max_ were linearly correlated to those of stomatal closure (Table 1). Heber et al. (1986) showed that photosynthetic rate decreased following stomatal closure after severing of a leaf [56]. Slightly shorter T_resp_(g_s_) than T_resp_(A) confirmed that the reduction of A_max_ was mediated by the response of g_s_. The slower reduction of A_max_ in injured leaves than in healthy leaves would increase carbon assimilation under water stress conditions and may be interpreted as a feedback mechanism to maximize photosynthesis under stress. However, severe O_3_ visible injury (>35%) shifted carbon sink to source because A_max_ was <0 µmol m^−2^ s^−1^ (Figure 3B).

At whole-tree level, the total carbon assimilation (A_tree_) and water loss (W_loss_) assessed under steady-state conditions were significantly lower in WAT trees exposed to ambient ozone than in EDU-protected trees in both September and October (Figure 5A–B). Such O_3_-induced reduction of photosynthesis and water loss was in agreement with meta-analysis results [36]. Dynamic and steady-state whole-tree WUEs showed a similar seasonal trend. WUE was significantly higher in EDU trees than in WAT trees, both in September and October and both when assessed under steady-state and dynamic conditions (Figure 5C and 6C). On average, WUE of trees exposed to ambient ozone was 2–4% lower than that of EDU-protected control trees in September and 6–8% lower in October. The decrease of tree-level WUE over time, in fact, was larger in WAT than in EDU trees, confirming the frequently reported decrease in leaf-level WUE in O_3_-exposed plants [33] and O_3_-injured leaves [41]. Also whole-tree dynamic carbon assimilation (A_tree_st_) and water loss (W_loss_st_) were significantly lower in WAT trees than in EDU-protected trees (Figure 6A–B). In contrast, ozone-induced stomatal sluggishness would be expected to increase whole-tree water loss. This response, however, was balanced by lower gas exchange (Figure 3) and premature shedding of injured leaves. After the onset of O_3_ visible injury in early September, ozone visible injury increased quickly (Figure 2B). In parallel, leaf abscission also progressed (Figure 2A), so that both whole-tree water loss and carbon assimilation were reduced. However, McLaughlin et al. (2007) reported that ambient O_3_ spikes significantly increased water loss of trees, as assessed from sap-flow measurements, suggesting that ozone-induced aberrations in the stomatal dynamics may differ depending on the species and the environmental conditions [32].

### Conclusions

One of the topical subjects in the assessment of O_3_ risk to forests is scaling up from leaf level to the stand and landscape level [4]. Further improvement of our understanding about stomatal responses to ambient O_3_ can be regarded as an essential factor in modelling and predicting forest responses to both O_3_ and climate [21]. Occurrence of O_3_ visible injury resulted in loss of stomatal control for water loss, but was compensated by lower stomatal conductance and premature leaf shedding. The resulting decline in whole tree ability of transpiring and sequestering atmospheric carbon is a significant effect of ambient ozone pollution.

Stomata play a crucial role in regulating plant gas exchange with the atmosphere, including O_3_ uptake [16]–[20]. Surface O_3_ concentrations are continuously increasing [4]. The climate change brings about the risk of drought and flooding [1]. The results of this study contribute new knowledge about water control and carbon sequestration of trees under ambient O_3_ exposure and suggest that the effects of O_3_–induced stomatal sluggishness on the whole-tree carbon and water balance are negligible.
